# Changes in device-matched peak inspiratory flow after bedside inhaler coaching and factors associated with residual suboptimal flow: a retrospective observational study

**DOI:** 10.3389/fphar.2026.1903204

**Published:** 2026-09-23

**Authors:** Yuhang Ding, Pengnan Xu, Yanhong Meng, Jinping Zhang, Qiuhui Wu

**Affiliations:** 1 Department of Pharmacy, Nanjing Drum Tower Hospital, China Pharmaceutical University, Nanjing, China; 2 Department of Pharmacy, Nanjing Drum Tower Hospital, Affiliated Hospital of Medical School, Nanjing University, Nanjing, China

**Keywords:** chronic airway disease, device resistance, hospitalized patients, inhaler devices, peak inspiratory flow training

## Abstract

**Background:**

Peak inspiratory flow rate (PIFR) is a critical determinant of inhaler-device compatibility, particularly when assessed under resistance conditions matching the prescribed inhaler. However, changes in device-matched PIFR patterns after inhaler coaching and factors associated with persistent abnormalities remain insufficiently characterized among hospitalized patients with chronic airway diseases.

**Methods:**

We conducted a retrospective single-center observational study of hospitalized adults with chronic obstructive pulmonary disease, asthma, or asthma–COPD overlap who underwent routine device-matched PIFR assessment between July 2024 and December 2025. PIFR was measured using the In-Check DIAL G16 before and after standardized bedside coaching under resistance settings matched to the prescribed inhaler. Logistic regression analyses were performed to identify factors associated with persistent post-coaching PIFR abnormalities.

**Results:**

Among 286 patients, the distribution of device-matched PIFR shifted substantially toward the device-specific optimal PIFR range after bedside assessment and coaching. The proportion of patients within the optimal PIFR range increased from 42.3% to 89.2%, whereas insufficient and excessive PIFR decreased from 25.2% to 6.9% and from 32.5% to 3.9%, respectively (all P < 0.001). Lower body mass index was independently associated with persistent insufficient PIFR, whereas ICS-containing therapy was also associated with this residual abnormality. Persistent excessive PIFR was uncommon and primarily observed among patients using pMDI/SMI devices.

**Conclusion:**

Device-matched PIFR assessment combined with individualized coaching was associated with a substantial shift toward the device-specific optimal PIFR range among hospitalized patients with chronic airway diseases. However, persistent insufficient PIFR remained in a subset of patients and was associated with lower BMI and ICS-containing therapy, suggesting that these factors may help identify patients who warrant closer reassessment of inhaler–device compatibility.

## 1 Introduction

Chronic obstructive pulmonary disease (COPD) and asthma are common chronic airway diseases for which inhaled therapy is central to long-term management. Current guidelines emphasize that the effectiveness of inhaled treatment depends not only on prescribing the appropriate medication, but also on selecting a device that matches the patient’s ability to use it correctly (GINA, 2025; GOLD, 2025). As inhaler options have expanded, the key clinical question has shifted from whether inhaled therapy is indicated to whether the chosen device can deliver medication effectively in an individual patient (Lavorini et al., 2014; Ohar et al., 2022; Mahler and Halpin, 2024; 2025).

Peak inspiratory flow rate (PIFR) is an important measure of inhalation capacity and device compatibility (Sanders, 2017; Ohar et al., 2022). Its clinical relevance, however, lies less in the absolute value than in whether an appropriate inspiratory flow can be achieved under the resistance conditions of a specific device. This is particularly relevant for dry powder inhalers (DPIs), which require sufficient inspiratory flow against device resistance to generate respirable particles, whereas pressurized metered-dose inhalers (pMDIs) and soft mist inhalers (SMIs) rely less on high inspiratory flow and more on coordinated inhalation technique (Ghosh et al., 2017; 2019; Haughney et al., 2021). Accordingly, recent reviews and methodological studies have emphasized that PIFR should, whenever possible, be assessed under device-matched resistance rather than inferred from general inspiratory parameters obtained from routine pulmonary function testing (Mahler, 2017; Usmani et al., 2018; Halpin and Mahler, 2024). The In-Check DIAL G16 is commonly used for this purpose because it can simulate the internal resistance of different inhalers and support repeat assessment under device-specific resistance settings (Barnes et al., 2020; Leving et al., 2022a).

Suboptimal PIFR is common in patients with chronic airway disease, particularly among hospitalized patients and those experiencing acute exacerbations, and has been associated with unfavorable clinical outcomes and increased healthcare utilization (Loh et al., 2017; Leving et al., 2022b; Mahler et al., 2022). Although PIFR-guided inhaler management and inhaler technique education may improve device compatibility, previous studies have primarily focused on stable COPD populations, single inhaler resistance settings, or DPI users assessed according to simplified threshold-based definitions of inadequate inspiratory flow (Chen et al., 2020; 2021). These limitations may restrict clinical applicability because hospitalized patients represent a more heterogeneous population with diverse chronic airway diseases, varying disease severity, and different inhaler-device requirements. Acute exacerbations induce dynamic changes in lung mechanics and inspiratory muscle function, which may render a previously adequate PIFR insufficient during hospitalization. In routine inpatient practice, clinicians frequently initiate, continue, or adjust inhaled therapies; however, the compatibility between the prescribed device and the patient’s inspiratory capability may not always be adequately evaluated. Importantly, clinical assessment should consider not only whether patients can generate sufficient inspiratory flow, but also whether their inspiratory pattern is appropriate for the resistance characteristics of the prescribed device. Therefore, evidence remains limited regarding whether hospitalized patients with different chronic airway diseases can achieve device-matched optimal PIFR after standardized coaching and which clinical characteristics are associated with persistent abnormalities in inspiratory flow performance.

Therefore, in this retrospective observational study, we aimed to characterize changes in device-matched PIFR before and after standardized bedside assessment and coaching among hospitalized patients with COPD, asthma, and asthma–COPD overlap. Unlike previous studies that predominantly evaluated stable COPD populations or single inhaler categories, our study included patients with multiple chronic airway diseases using different inhaler devices, with PIFR assessed under resistance conditions matched to the prescribed inhaler. Furthermore, we explored clinical characteristics associated with persistent post-coaching PIFR abnormalities, including insufficient and excessive PIFR, to identify patient subgroups who may require additional assessment of inhaler-device compatibility. These findings may provide further insights into individualized inhaler management and the potential role of device-matched PIFR reassessment in inpatient respiratory care.

## 2 Methods

### 2.1 Study design and setting

This retrospective single-center observational study was conducted at the Department of Respiratory Medicine, Jiangbei Campus of Nanjing Drum Tower Hospital, China. The study included patients admitted to the respiratory ward who underwent inpatient inhaler assessment and received pharmacist-delivered coaching during hospitalization. Consecutive hospitalization records from July 2024 through December 2025 were screened using routine clinical records and chart review. The unit of analysis was the individual patient. For patients with more than one eligible hospitalization or repeated PIFR assessments during the study period, only the first eligible hospitalization with complete paired pre- and post-assessment PIFR measurements under the prescribed device-matched resistance was included in the primary analysis. For patients prescribed more than one maintenance inhaler during the index hospitalization, the first maintenance inhaler prescribed by the treating physician was selected according to a predefined study criterion that reflected the routine clinical workflow. PIFR was assessed under the resistance setting matched to this inhaler to evaluate the compatibility between the patient’s inspiratory capacity and the prescribed device. Evaluating a single device per patient avoided intra-individual clustering, ensured independence of observations in the statistical analyses, and provided a consistent approach for inhaler selection across all patients.

The study was approved by the Ethics Committee of Nanjing Drum Tower Hospital (Approval No.:2026-0280) and conducted in accordance with the Declaration of Helsinki. Given the retrospective nature of the study and the exclusive use of anonymized data obtained from routine clinical practice, the requirement for written informed consent was waived by the ethics committee. Clinical data access was obtained through institutional approval procedures, and only authorized study investigators accessed the anonymized records.

### 2.2 Participants

Adults aged ≥18 years were eligible if they had a documented diagnosis of COPD, asthma, or asthma-COPD overlap (ACO); received inhaled therapy during hospitalization; and had complete paired pre- and post-assessment PIFR measurements obtained under the resistance setting matched to the prescribed inhaler. COPD and asthma diagnoses were established in routine clinical practice according to GOLD (2025) and GINA (2025), respectively. Patients were excluded if impaired mental status, cognitive dysfunction, or poor cooperation precluded assessment; if no documented inhaler regimen was available; or if paired PIFR data for the index hospitalization were incomplete.

### 2.3 Data collection and clinical definitions

Baseline variables included age, sex, body mass index (BMI), smoking history, primary respiratory diagnosis, prescribed inhaled medications, inhaler device type, PIFR measurements, emergency department admission before hospitalization, systemic corticosteroid use, comorbidities, length of hospital stay, and the number of acute exacerbations in the previous year.

Comorbidities were defined as the total number of documented chronic comorbid conditions recorded in the electronic medical record at the index hospitalization. Hospitalization status was categorized as non-acute, mild acute exacerbation (AE), or severe AE. Non-acute referred to hospitalization during a clinically stable phase of chronic airway disease. Mild AE referred to hospitalization for an acute exacerbation without escalation to high-flow nasal cannula, noninvasive ventilation, invasive mechanical ventilation, or ICU transfer. Severe AE referred to acute exacerbation requiring escalation of respiratory support or ICU transfer. This classification was adapted from guideline-based assessments of exacerbation severity, particularly the need for hospitalization, respiratory failure management, and escalation of respiratory support (Rochwerg et al., 2017; GOLD, 2025). The number of acute exacerbations during the previous year was retrospectively extracted from electronic medical records and previous outpatient and hospitalization records. Acute exacerbations were identified according to the definitions provided in the GOLD and GINA (GINA, 2025) guidelines, which characterize exacerbations as episodes of acute worsening of respiratory symptoms requiring additional therapy. For this study, an exacerbation event was recorded when it resulted in treatment escalation, including the use of systemic corticosteroids and/or antibiotics, an emergency department visit, or hospitalization related to respiratory deterioration.

### 2.4 PIFR assessment

PIFR was measured using the In-Check DIAL® G16, a low-range inspiratory flow meter capable of simulating device-specific inhaler resistance, with a measurement range of 15–120 L/min and a stated accuracy of ±10% or 10 L/min (Sanders, 2017). Resistance settings were categorized as R0, R1, R2, or R3 according to the prescribed inhaler and study protocol. Before each measurement, the resistance dial was adjusted to match the internal resistance of the patient’s prescribed inhaler, enabling assessment of PIFR under device-matched resistance conditions. Measurements were performed with patients in the seated position. According to the manufacturer’s instructions, participants were instructed to exhale gently to functional residual capacity or residual volume before each inspiratory maneuver. Participants were then asked to inhale through the In-Check DIAL “as if using your inhaler.” Each participant performed three inspiratory maneuvers, with approximately 30–60 s of rest between attempts, and the highest acceptable PIFR was used for analysis.

Using prespecified device-specific criteria (Supplementary Table S1) (Ghosh et al., 2017; Respiratory Therapy and Pulmonary Function Group of the Respiratory Disease Specialty Branch, Shanghai Medical Association, (2023) PIFR was descriptively classified as insufficient, optimal, or excessive according to whether the measured value was below, within, or above the device-specific optimal PIFR range. Patients whose measured PIFR fell outside the device-specific optimal PIFR range received standardized individualized coaching delivered by trained respiratory pharmacists immediately after the initial assessment. All pharmacists involved in the study received prior training on In-Check DIAL G16 operation, inhalation maneuver instruction, and PIFR interpretation. The coaching procedure included three standardized steps: (1) explanation of the target inspiratory maneuver according to the prescribed inhaler resistance; (2) pharmacist demonstration of the recommended inhalation pattern; and (3) patient practice with immediate verbal feedback. Patients with insufficient PIFR were instructed to generate a faster and more forceful inspiratory maneuver, whereas patients with excessive PIFR were instructed to perform a slower and more controlled inhalation. The coaching process generally lasted approximately 5–10 min, after which PIFR was reassessed using the same resistance setting. As recommended by previous studies, each participant performed three inspiratory maneuvers, and the highest PIFR value was recorded for analysis (Jailaini et al., 2024; Pankovitch et al., 2025).

For regression analyses, post-training PIFR abnormalities were defined based on the device-specific optimal PIFR ranges. Persistent insufficient PIFR was defined as a post-training PIFR value below the lower limit of the corresponding device-specific optimal PIFR range. Persistent excessive PIFR was defined as a post-training PIFR value above the upper limit of the corresponding device-specific optimal PIFR range. Because insufficient and excessive PIFR may represent distinct physiological and behavioral mechanisms, these two outcomes were analyzed separately.

### 2.5 Statistical analysis

Continuous variables were presented as mean ± SD or median (IQR), as appropriate, and categorical variables as number (percentage). Only patients with complete paired pre- and post-training PIFR measurements and a documented inhaler regimen were included in the analytic cohort. There were no missing data among included patients for variables used in the primary analyses; therefore, no imputation was performed.

Pre- and post-training PIFR were compared within each resistance category (R0-R3) using paired t tests or Wilcoxon signed-rank tests, as appropriate. Changes in each PIFR category (insufficient, optimal, and excessive) before and after inhaler technique training were assessed using McNemar tests for paired binary comparisons.

Because persistent insufficient and persistent excessive PIFR may reflect different mechanisms, factors associated with these two post-training outcomes were evaluated separately. For persistent insufficient PIFR, univariable logistic regression analyses were initially performed to explore associations between baseline clinical characteristics and the outcome. Candidate covariates were prespecified based on clinical relevance and previously reported factors potentially associated with inspiratory flow performance. These variables included sex, age, body mass index (BMI), primary diagnosis, severity of acute exacerbation, emergency department admission, comorbidities, number of acute exacerbations during the previous year, smoking status, and use of ICS-containing regimens.

Due to the limited number of patients with persistent insufficient PIFR after training, the multivariable model was restricted to two variables to minimize the risk of overfitting. Candidate variables were initially evaluated using univariable logistic regression analyses, and variables with a P value < 0.05 were considered for inclusion in the multivariable logistic regression model. The selected covariates were both statistically significant in univariable analyses and clinically plausible based on previous evidence. Multicollinearity was assessed using the variance inflation factor (VIF), and all included variables showed VIF values <5. Model calibration was evaluated using the Hosmer–Lemeshow goodness-of-fit test, and model explanatory performance was assessed using the Nagelkerke *R* ^2^ statistic. Adjusted odds ratios (ORs) and 95% confidence intervals (CIs) were reported.

Because persistent excessive PIFR occurred infrequently after training, exploratory analyses were performed without multivariable modeling. Baseline characteristics between patients with and without persistent excessive PIFR were compared using the Mann–Whitney U test for continuous variables and Fisher’s exact test for categorical variables, as appropriate. All tests were 2-sided, and P < 0.05 was considered statistically significant. Analyses were performed using SPSS version 27.0, GraphPad Prism version 10.0, and Stata version 17.0.

## 3 Results

### 3.1 Demographic characteristics of participants

Of 298 hospitalization records screened, 286 unique patients were included in the final analysis after exclusion of records not meeting prespecified analytic criteria. Baseline characteristics are summarized in Table 1. Participants were older (median age, 73 years (IQR, 67–77)), predominantly male (76.9%), and primarily diagnosed with COPD (84.6%). Most assessments were performed during acute hospitalization, including 56.9% with mild acute exacerbation and 11.3% with severe acute exacerbation (Table 1). Dry powder inhalers were the most prescribed devices (64.3%). Across resistance settings, measurements were concentrated in the lower to intermediate resistance categories, particularly R1 and R2 devices. Among DPI users, R2 was the most frequent resistance class (47.2%), followed by R1 (40.2%) and R3 (12.6%) (Figure 1).

**TABLE 1:** participant characteristics at time of peak inspiratory flow measurement.

Characteristic	Value
Demographics	
Participants (n)	286
Age (years)	73 (67.77)
Male: Female (n)	220:66
BMI (kg/m^2^)	22.28 (19.66.24.68)
Diagnosis (no. (%))	
COPD	242 (84.6)
Asthma	34 (11.8)
ACO	10 (3.6)
Severity of acute exacerbation (no. (%))	
Non-acute	91 (31.8)
Mild AE	163 (56.9)
Severe AE	32 (11.3)
ED admission (no. (%))	115 (40.2)
Systemic corticosteroid use (no. (%))	110 (38.4)
Comorbidities	7 (5, 9)
LOS (days)	7 (5, 9)
Number of acute exacerbations in the past year	
0 (%)	83 (29.0)
1 (%)	97 (33.9)
≥2 (%)	106 (37.1)
Smoking status	
Current (no. (%))	45 (15.7)
Former (no. (%))	141 (49.4)
Never (no. (%))	100 (34.9)
Smoking pack-year history (year)	40 (30.50)
Prior inhaler use (no. (%))	164 (57.3)
Device type	
DPI	184 (64.3)
pMDI/SMI	102 (35.7)
Resistance class	
R0/R1/R2/R3	102/74/87/23

Data are expressed as n (%) or median (IQR) unless otherwise stated. BMI, body mass index; COPD, chronic obstructive pulmonary disease; ACO, asthma–COPD overlap; AE, acute exacerbation; ED, emergency department; LOS, length of stay; DPI, dry powder inhaler; pMDI, pressurized metered-dose inhaler; SMI, soft mist inhaler; R0–R3, resistance classes used for peak inspiratory flow measurement. Severity categories were defined as “Nonacute” (non-acute hospitalization), “Mild AE” (acute phase without escalation of respiratory support), and “Severe AE” (acute phase with escalation of respiratory support or ICU transfer). Prior inhaler use was defined as documented use of any inhaled medication delivered via a DPI, pMDI, or SMI before the index hospitalization.

**FIGURE 1:**
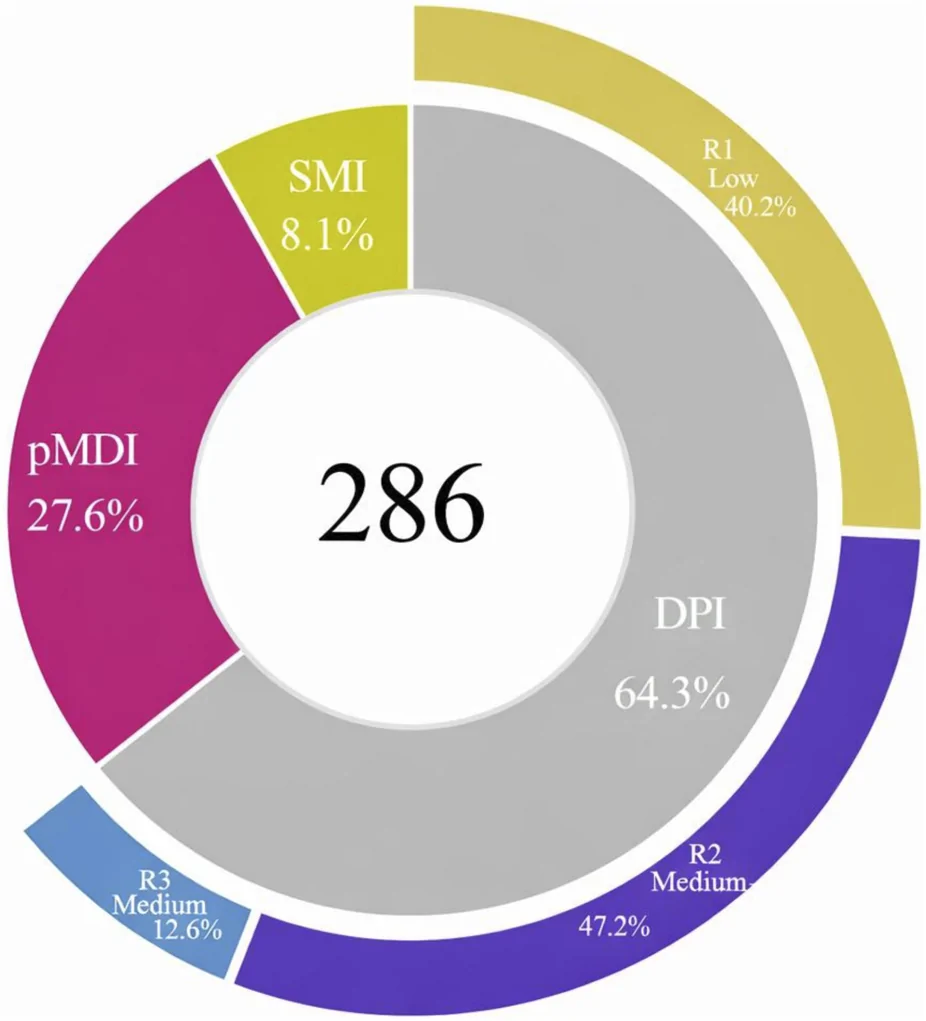
Distribution of inhaler device types and resistance classes at the study visit.

### 3.2 PIFR changes after training

A total of 286 patients were included in the paired analysis of PIFR before and after training. Following training, PIFR demonstrated substantial resistance-specific changes rather than a uniform increase across all settings. Compared with pre-training values, mean PIFR decreased by 20.25 L/min (95% CI, 14.85–25.65) under the R0 setting, whereas it increased by 7.05 L/min (95% CI, 3.35–10.75), 11.35 L/min (95% CI, 8.47–14.23), and 15.17 L/min (95% CI, 9.50–20.84) under the R1, R2, and R3 settings, respectively (all P < 0.001). In parallel, the overall distribution of PIFR levels shifted markedly toward the device-specific optimal PIFR range after training. Category-specific paired binary comparisons showed that the proportion of patients with insufficient PIFR decreased from 25.2% to 6.9%, the proportion with excessive PIFR decreased from 32.5% to 3.9%, and the proportion with optimal PIFR increased from 42.3% to 89.2% (all McNemar P < 0.001) (Table 2). These findings indicate that bedside coaching was associated with a shift toward device-specific optimal PIFR ranges across different inhaler resistance settings.

**TABLE 2:** Distribution and levels of PIFR before and after training in the study patients.

Variable	Pre-PIFR	Post-PIFR	Mean change (95% CI)	Effect size (Cohen’s dz)	t	*P*-value
Resistance class						
R0	50.79 ± 27.65	30.54 ± 2.71	−20.25 (−25.65, −14.85)	−0.73	−7.443	<0.001
R1	39.42 ± 18.96	46.47 ± 9.20	7.05 (3.35.10.75)	0.44	3.802	<0.001
R2	40.94 ± 16.98	52.30 ± 12.50	11.35 (8.47, 14.23)	0.83	7.381	<0.001
R3	32.65 ± 14.70	47.83 ± 11.48	15.17 (9.50.20.84)	1.15	5.549	<0.001
The level of PIFR						
Insufficient	72 (25.2)	20 (6.9)	-	-	-	<0.001
Optimal	121 (42.3)	255 (89.2)	-	-	-	<0.001
Excessive	93 (32.5)	11 (3.9)	-	-	-	<0.001

Data are presented as mean ± SD for continuous variables and n (%) for categorical variables. PIFR, peak inspiratory flow rate. Pre- and post-training PIFR indicate measurements obtained before and after training, respectively. R0–R3 denote the resistance classes used for PIFR assessment. Insufficient, optimal, and excessive PIFR were classified according to device-specific optimal PIFR ranges. P values for insufficient, optimal, and excessive PIFR were derived from category-specific McNemar tests, with each category analyzed separately as a paired binary outcome. Mean change was calculated as post-training PIFR minus pre-training PIFR. Effect sizes were calculated using paired Cohen’s dz.

Violin and Sankey plots showed a clear resistance-dependent transition pattern after training. Under the R0 setting, PIFR shifted predominantly from excessive toward optimal, whereas under the R1-R3 settings, the dominant transition was from insufficient toward optimal. These resistance-specific flow patterns visually reinforced the overall shift toward the device-specific optimal PIFR range after training (Figures 2, 3). Similar patterns were observed across clinical subgroups, with a general shift toward the device-specific optimal PIFR category after coaching (Supplementary Figure S1).

**FIGURE 2:**
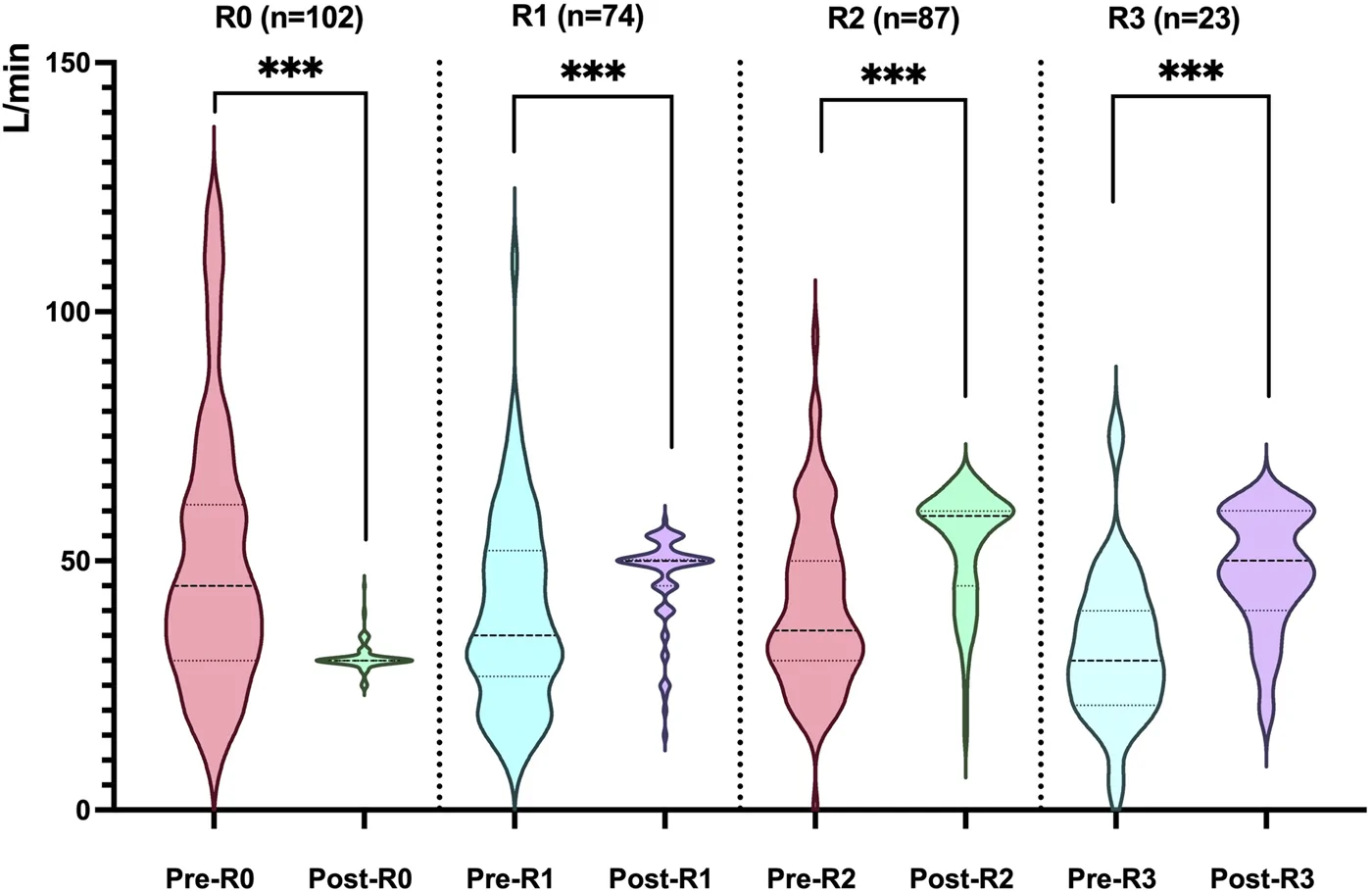
Distribution of peak inspiratory flow rate before and after coaching across different inhaler resistance settings. Notes: The number of observations (n) within each resistance class is indicated above the corresponding groups. Violin plots show the distribution of PIFR before and after coaching. P values were derived from paired comparisons of pre- and post-coaching PIFR measurements.

**FIGURE 3:**
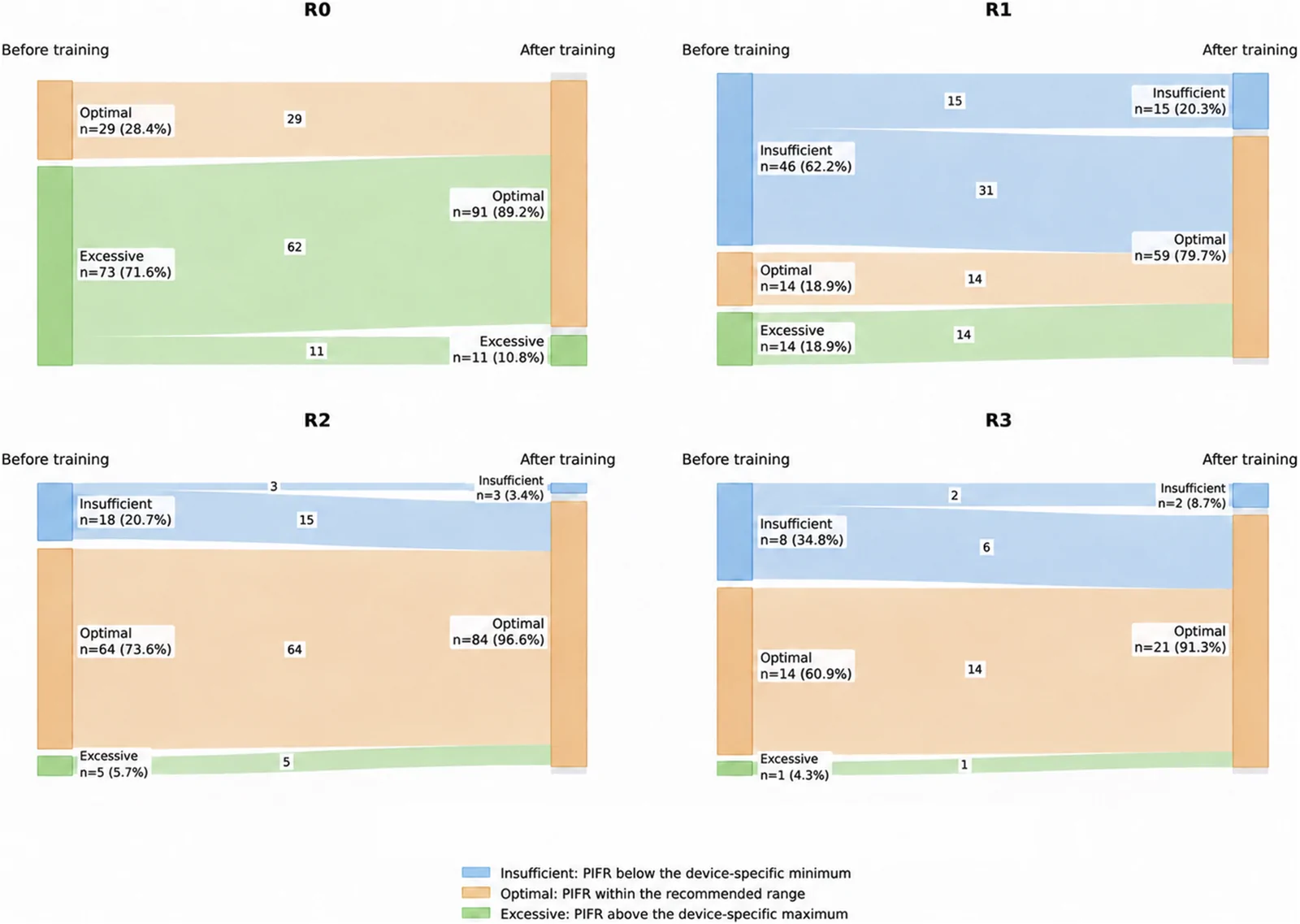
Transitions in peak inspiratory flow rate categories before and after coaching across inhaler resistance classes. The number of observations (n) is indicated for each PIFR category and transition. The width of each flow represents the number of observations transitioning between PIFR categories. Transitions represent changes from insufficient, optimal, or excessive PIFR categories following standardized coaching.

### 3.3 Factors associated with persistent insufficient PIFR after training

Among the 286 participants included in the paired analysis, 20 patients (7.0%) had persistent insufficient PIFR after inhaler training. In univariable logistic regression analyses, lower BMI was significantly associated with persistent insufficient PIFR after training (OR, 0.851; 95% CI, 0.752–0.963; P = 0.010). In addition, the use of ICS-containing therapy was associated with persistent insufficient PIFR (OR, 6.852; 95% CI, 2.409–19.488; P < 0.001). Other evaluated variables, including age, sex, primary diagnosis, acute exacerbation severity, emergency department admission, comorbidity burden, smoking status, and previous-year exacerbation frequency, were not significantly associated with persistent insufficient PIFR (Table 3).

**TABLE 3:** Factors associated with persistent insufficient peak inspiratory flow rate after inhaler training.

Risk factor	Univariate analysis		Multivariate analysis	
	OR (95% CI)	p-value	OR (95% CI)	p-value
Sex	2.391 (0.933.6.125)	0.069	**-**	**-**
Age (years)	1.018 (0.965.1.073)	0.516	-	-
BMI	**0.851 (0.752,0.963)**	**0.010**	**0.872 (0.770,0.988)**	**0.031**
Diagnosis (COPD vs. Others)	0.273 (0.036.2.093)	0.212	-	-
Severity of acute exacerbation				
Mild AE vs. non-acute	1.367 (0.466.4.010)	0.569	-	-
Severe AE vs. non-acute	1.779 (0.400.7.910)	0.449	**-**	**-**
ED admission	1.533 (0.617.3.811)	0.357	-	-
Comorbidities	1.005 (0.884.1.143)	0.940	-	-
Past-year AEs, n	1.157 (0.827.1.618)	0.394	**-**	**-**
Smoking status	​	​	​	-
Former vs. Never	0.511 (0.205.1.274)	0.150	-	-
ICS-containing, yes vs. no	**6.852 (2.409,19.488)**	**<0.001**	**6.202 (2.157,17.832)**	**<0.001**

OR, odds ratio; CI, confidence interval; BMI, body mass index; COPD, chronic obstructive pulmonary disease; ICS, inhaled corticosteroid. Past-year AEs, number of acute exacerbations in the previous year. Non-acute: Non-acute hospitalization; AE: acute exacerbation; Mild AE: Mild acute exacerbation; Severe AE: Severe acute exacerbation; DPI: dry powder inhaler; pMDI: pressurized metered-dose inhaler; SMI: soft mist inhaler; ED: emergency department. Comorbidities represent the total number of documented chronic comorbid conditions.

Bold values indicate statistically significant results (P < 0.05).

In the adjusted analysis, lower BMI remained independently associated with persistent insufficient PIFR after training (adjusted OR, 0.872; 95% CI, 0.770–0.988; P = 0.031). Similarly, ICS-containing therapy remained significantly associated with persistent insufficient PIFR (adjusted OR, 6.202; 95% CI, 2.157–17.832; P < 0.001). The Hosmer–Lemeshow goodness-of-fit test indicated acceptable model calibration (χ^2^ = 3.785, P = 0.876), and the Nagelkerke R^2^ was 0.173.

### 3.4 Characteristics of patients with persistent excessive PIFR

Persistent excessive PIFR after inhaler training was observed in 11 of 286 patients (3.8%). Compared with patients without persistent excessive PIFR, patients with persistent excessive PIFR showed marked differences in inhaler device type. All patients with persistent excessive PIFR were using pMDI/SMI devices, whereas no cases were observed among DPI users (100.0% vs. 33.1%, Fisher’s exact test, P < 0.001).

A higher proportion of patients with severe acute exacerbations was observed among those with persistent excessive PIFR (27.3% vs. 10.5%, P = 0.034). Other baseline characteristics, including age, BMI, sex, diagnosis, ED admission, comorbidity burden, smoking status, ICS-containing therapy, and previous-year exacerbation frequency, were not significantly different between groups (all P > 0.05) (Supplementary Table S2).

## 4 Discussion

In this retrospective pre–post observational study of hospitalized patients with chronic airway diseases, device-matched PIFR shifted substantially toward the device-specific optimal PIFR range after bedside assessment and standardized coaching. The proportion of patients within the optimal range increased from 42.3% to 89.2%, while insufficient and excessive PIFR decreased to 6.9% and 3.9%, respectively. These findings indicate that, for many hospitalized patients, initially suboptimal device-matched PIFR was followed by a shift toward the device-specific optimal PIFR range after individualized feedback and repeat assessment. However, a small proportion of patients continued to demonstrate persistent PIFR abnormalities despite coaching, and specific patient and device characteristics were associated with these residual abnormalities, suggesting that the response to inhaler coaching may vary among patients. These findings support the potential role of incorporating device-matched PIFR assessment and structured coaching into inpatient inhaler management.

A key observation of this study was that bedside coaching was associated with resistance-specific changes in PIFR rather than a uniform increase in inspiratory flow. The greatest increases were observed under the higher resistance settings (R2 and R3), with mean increases exceeding 10 L/min, whereas PIFR decreased under the R0 setting. This pattern suggests that coaching primarily modified the inspiratory maneuver toward device-compatible flow characteristics, rather than simply promoting higher peak flow. These findings highlight that optimal inhalation performance should not be defined by achieving the highest possible PIFR, but rather by achieving an appropriate inspiratory profile that matches device requirements. Excessively high PIFR may also be undesirable, as excessively rapid inhalation can increase oropharyngeal deposition and potentially reduce the proportion of medication reaching the lower airways. This issue may be particularly relevant for devices such as pMDIs and SMIs, where inspiratory flow control and coordination play an important role. Conversely, insufficient PIFR may impair aerosol generation and drug delivery. Therefore, PIFR-guided inhalation training, including visual feedback to help patients adjust their inspiratory effort, represents an approach not only to identify suboptimal inhalation patterns but also to guide patients toward a more appropriate inhalation maneuver (Mahler et al., 2014; 2019).

Such resistance-dependent changes further underscore the importance of assessing PIFR under device-matched conditions when evaluating inhaler suitability. Different inhaler platforms require different inspiratory strategies: DPIs rely on sufficient inspiratory flow to achieve effective powder deaggregation and dispersion (Ghosh et al., 2017; Ohar et al., 2022; Mahler and Halpin, 2024), whereas pMDIs and SMIs depend more on inspiratory timing, flow profile, and coordination (Anderson et al., 2022; Suter et al., 2025). Given that suboptimal PIFR has been associated with poorer clinical outcomes, including increased exacerbation risk and reduced treatment response in COPD (Chen et al., 2021; Mahler et al., 2023), device-matched PIFR assessment may provide clinically relevant information for characterizing inhaler–device compatibility and identifying patients with PIFR outside the device-specific optimal range. However, whether PIFR-guided reassessment should inform inhaler selection or device modification requires confirmation in prospective studies.

Although previous studies have characterized suboptimal PIFR and highlighted the importance of inhaler technique assessment (Meng et al., 2024; Perugini et al., 2025), persistent insufficient and excessive PIFR after coaching have received limited attention. Most previous investigations have focused on inadequate PIFR defined by a single threshold, particularly among stable COPD populations using dry powder inhalers (Loh et al., 2017; Leving et al., 2022b; Mahler and Halpin, 2025). In contrast, our study separately characterized persistent insufficient and excessive PIFR after standardized coaching, showing that 6.9% of patients remained insufficient and 3.9% exhibited excessive inspiratory flow. Furthermore, our hospitalized cohort included patients with COPD, asthma, and asthma–COPD overlap using DPI, pMDI, and SMI devices, with PIFR assessed under resistance conditions matched to the prescribed inhaler. These findings suggest that residual inspiratory flow abnormalities after coaching may represent different clinical patterns rather than a single limitation of inspiratory capacity.

Lower BMI was independently associated with persistent insufficient PIFR after bedside coaching, suggesting that patients with lower BMI may be more likely to remain below the device-specific optimal PIFR range after coaching. Generating an appropriate inspiratory maneuver likely depends not only on understanding instructions, but also on inspiratory muscle reserve, nutritional status, frailty, and overall functional capacity, which may affect the ability to generate and reproduce adequate flow under device-matched resistance conditions (Quanjer et al., 2012; Stanojevic et al., 2022; Trushenko et al., 2025). Previous studies have similarly linked low body weight, reduced functional status, and greater COPD-related disease burden with lower PIFR or reduced suitability for flow-dependent inhaler devices (Leving et al., 2022b; Ohar et al., 2022; Trushenko et al., 2025). In our cohort, each increase in BMI was associated with lower odds of persistent insufficient PIFR, indicating that patients with lower BMI may remain vulnerable to inadequate inspiratory flow even after standardized bedside coaching. These findings suggest that patients with lower BMI may represent a subgroup in whom repeat device-matched PIFR assessment and closer evaluation of inhaler–device compatibility warrant further investigation.

ICS-containing therapy was also independently associated with persistent insufficient PIFR after bedside coaching. This finding should not be interpreted as evidence that ICS therapy directly impairs inspiratory flow. Rather, ICS-containing regimens may serve as a marker of underlying clinical characteristics, including greater disease burden, more frequent exacerbations, or asthma-related/eosinophilic features, in which ICS use is more likely to be considered (Quint et al., 2023; Chinese Thoracic Society Chinese Medical Association, 2025; GOLD, 2025). However, the present study did not directly evaluate the mechanisms underlying this association. The mechanisms therefore remain uncertain, and the observed association should be interpreted cautiously.

Persistent excessive PIFR after coaching was uncommon but demonstrated a distinct pattern from persistent insufficient PIFR. Notably, in our cohort, all patients with persistent excessive PIFR were using pMDI/SMI devices, whereas no such cases were observed among DPI users, suggesting a potential device-related pattern that warrants further investigation. This finding may be clinically relevant because pMDIs and SMIs generally benefit from a slower and more controlled inhalation maneuver, and persistently excessive inspiratory flow may suggest that some patients have not fully adapted their inspiratory pattern to device requirements despite bedside instruction (Lavorini et al., 2014; Usmani et al., 2018; Anderson et al., 2022). Therefore, persistent excessive PIFR should not simply be interpreted as better inspiratory capacity, but rather as another potential form of device–patient mismatch. Whether additional inhaler technique assessment, repeated coaching, or visual feedback improves alignment with device-specific PIFR requirements requires prospective evaluation.

Several limitations should be considered when interpreting these findings. This retrospective single-center study included a predominantly COPD population, which may limit the generalizability of the results to other respiratory diseases or clinical settings. Although multivariable analyses were performed, residual confounding cannot be excluded, as some factors potentially influencing inhaler performance, including lung function, inspiratory muscle strength, cognitive ability, and physical function, were not available for analysis. In addition, the relatively small number of outcome events for persistent insufficient PIFR may have affected model stability and should be considered when interpreting the regression results. PIFR was assessed immediately after bedside coaching during hospitalization; therefore, whether these changes are sustained after discharge or translate into improvements in long-term inhaler technique and clinical outcomes remains unknown. Furthermore, PIFR was defined as the highest value among three repeated maneuvers, reflecting maximal inspiratory capacity under standardized conditions. However, this approach may overestimate habitual inspiratory performance in patients using inhalers requiring lower inspiratory effort, which warrants further evaluation under real-world conditions. Finally, the infrequent occurrence of persistent excessive PIFR may have limited statistical power. These observations should be considered hypothesis-generating and require confirmation in prospective multicentre studies. Future multicentre studies with controlled designs and longitudinal follow-up are warranted to further evaluate the clinical utility of device-matched PIFR reassessment.

These findings suggest that device-matched PIFR assessment may provide additional information for identifying patients with potential inhaler–device mismatch. Bedside assessment and coaching were associated with an overall shift toward the device-specific optimal PIFR range; however, a subset of patients continued to demonstrate insufficient PIFR after coaching, particularly those with lower BMI and those receiving ICS-containing regimens. These observations should be considered hypothesis-generating and require confirmation in prospective multicentre studies. Such studies are also needed to determine the clinical utility of PIFR-guided assessment and whether related interventions improve clinical outcomes.

## Data Availability

The datasets analyzed in this study are not publicly available due to institutional data-protection requirements concerning clinical data. Data may be made available from the corresponding authors upon reasonable request and subject to approval by Nanjing Drum Tower Hospital.
