# Supplementary material for: Changes in device-matched peak inspiratory flow after bedside inhaler coaching and factors associated with residual suboptimal flow: a retrospective observational study
Source: Front Pharmacol. 2026 Sep 23;17:1903204. doi: 10.3389/fphar.2026.1903204 (PMC13642123; doi:10.3389/fphar.2026.1903204)
Supplement: Supplementary file 1 [file Supplementaryfile1.docx]

Supplementary Material

**Supplementary Table S1. Device-specific target ranges used for classification of pre-training and post-training peak inspiratory flow rate (PIFR)**

| **Device class** | **Resistance level** | **Optimal PIFR range used in this study (L/min)** | **Classification rule** |
| --- | --- | --- | --- |
| pMDI/SMI | R0 | 10~30 | Insufficient:<10 Optimal:10~30; Excessive:>30 |
| DPI (simulating Breezhaler®) | R1 | 50 | Insufficient:<50 Optimal:50; Excessive:>50 |
| DPI (simulating Accuhaler®, Diskhaler®, Ellipta®) | R2 | 30~60 | Insufficient:<30 Optimal:30~60; Excessive:>60 |
| DPI (simulating Turbohaler®) | R3 | 30~60 | Insufficient:<30 Optimal:30~60; Excessive:>60 |

Abbreviations: PIFR, peak inspiratory flow rate; pMDI, pressurized metered-dose inhaler; DPI, dry powder inhaler; SMI, soft mist inhaler.
Note: Different inhalers have different minimum and ideal inspiratory flow requirements; for pMDIs, a slower and more stable inspiratory pattern is recommended, with an optimal flow around 30 L/min in adults.

**Supplementary Figure S1. Peak inspiratory flow rates (PIFR) before and after bedside coaching in overall patients and subgroups stratified by hospitalization status and primary diagnosis.**

**Supplementary Table S2. Exploratory comparison of baseline characteristics between patients with and without persistent excessive PIFR after inhaler training**

| **Characteristic** | **No persistent excessive PIFR (n=275)** | **Persistent excessive PIFR (n=11)** | **P** |
| --- | --- | --- | --- |
| **Gender, n (%)** |  |  | 0.466 |
| Male | 210 (76.3) | 10 (90.9) |  |
| Female | 65 (23.7) | 1 (9.1) |  |
| **Age (years)** | 73 (67,77) | 73 (70,77) | 0.556 |
| **BMI, kg/m²** | 22.28 (19.94,24.68) | 22.28 (16.77,23.15) | 0.131 |
| **Diagnosis, n (%)** |  |  | 0.384 |
| COPD | 234 (85.1) | 8 (72.7) |  |
| Others | 41 (14.9) | 3 (27.3) |  |
| **Severity of Acute Exacerbation** |  |  | **0.034** |
| Non‑acute | 91 (33.1) | 0 (0) |  |
| Mild AE | 155 (56.4) | 8 (72.7) |  |
| Severe AE | 29 (10.5) | 3 (27.3) |  |
| **ED admission, n (%)** | 109 (39.6) | 6 (54.5) | 0.359 |
| **Comorbidities, median (IQR)** | 7 (5,9) | 8 (5,11) | 0.319 |
| **Past‑year AEs, median (IQR)** | 1 (0,2) | 2 (1,3) | 0.107 |
| **Current smoking, n (%)** |  |  | 1.000 |
| Yes | 44 (16.0) | 1 (9.1) |  |
| No | 231 (84.0) | 10 (90.9) |  |
| **ICS-containing (yes), n (%)** | 181 (65.8) | 9 (81.8) | 0.345 |
| **Device type, n (%)** |  |  | **<0.001** |
| DPI | 184 (66.9) | 0 (0) |  |
| pMDI/SMI | 91 (33.1) | 11 (100.0) |  |

*Notes: BMI, body mass index; COPD, chronic obstructive pulmonary disease; ICS, inhaled corticosteroid. Past-year AEs, number of acute exacerbations in the previous year. Non-acute: Non-acute hospitalization; AE: acute exacerbation; Mild AE: Mild acute exacerbation; Severe AE：Severe acute exacerbation; DPI: dry powder inhaler; pMDI: pressurized metered-dose inhaler; SMI: soft mist inhaler; ED: emergency department.*
